# Enhancing the Stability of Litsea Cubeba Essential Oil Emulsions Through Glycosylation of Fish Skin Gelatin via Dry Maillard Reaction

**DOI:** 10.3390/foods13233847

**Published:** 2024-11-28

**Authors:** Naiwen Xing, Shikang Tang, Xuejiao Wang, Chaofan Guo, Xiaosong Hu, Junjie Yi

**Affiliations:** 1 Faculty of Food Science and Engineering, Kunming University of Science and Technology, Kunming 650500, China; m18846062506@163.com (N.X.); 15969040899@163.com (S.T.); wangxuejiao173@hotmail.com (X.W.); huxiaos@263.net (X.H.); yjjpp@163.com (J.Y.); 2 International Green Food Processing Research and Development Center of Kunming City, Kunming 650500, China; 3 Yunnan Key Laboratory of Plateau Food Advanced Manufacturing, Kunming 650500, China; 4 College of Food Science and Nutritional Engineering, China Agricultural University, Beijing 100083, China

**Keywords:** dry Maillard reaction, fish skin gelatin, Litsea cubeba oil, nanoemulsion stability

## Abstract

Emulsions are widely utilized in food systems but often face stability challenges due to environmental stresses, such as pH, ionic strength, and temperature fluctuations. Fish skin gelatin (FSG), a promising natural emulsifier, suffers from limited functional properties, restricting its broader application. This study explored the enhancement of emulsion stability in Litsea cubeba essential oil systems through the glycosylation of fish skin gelatin (FSG) with dextran via the dry Maillard reaction. Among dextrans of varying molecular weights (10 kDa, 100 kDa, 200 kDa, and 500 kDa), the 200 kDa dextran exhibited the best emulsification performance, achieving a remarkable 160.49% increase in stability index. The degree of grafting (DG) increased with molecular weight, peaking at 34.77% for the 500 kDa dextran, followed by 23.70% for the 200 kDa variant. The particle size of the FSG–Dex 200 kDa conjugate emulsion was reduced to 639.1 nm, compared to 1009–1146 nm for the unmodified FSG, while hydrophobicity improved by 100.56%. The zeta potential values approached 30 mV, indicating enhanced stability. Furthermore, glycosylation significantly improved antioxidant activity, as evidenced by increased radical scavenging capacity in both DPPH and ABTS assays. These findings underscore the potential of glycosylated FSG as a natural emulsifier in food applications.

## 1. Introduction

Gelatin is a naturally occurring amphiphilic macromolecular material, and marine resources provide an important source for its production. In particular, the by-products of fish processing and the waste generated are excellent sources for the production of gelatin. This also solves the problem of certain religious people—Muslims and Jews, for example—do not accept items made from animal gelatin [1,2]. Fish skin gelatin (FSG) is recognized for its high quality and is widely used in the food, pharmaceutical, materials, and chemical industries [3]. Its high surface activity allows FSG to act as an emulsifier in oil-in-water emulsions, helping to mitigate issues related to chemical oxidation, taste, and odor. Additionally, [(Litsea cubeba (Lour.) Pers.] essential oil, a natural spice oil extracted from the fruit of the Litsea cubeba plant, has garnered attention for its unique aroma and flavor [4]. In culinary applications, particularly in seafood dishes, Litsea cubeba oil effectively enhances the overall taste and texture of fish, making it a valuable ingredient in the preparation of dishes like grilled fish [5]. This highlights its significant potential in utilizing marine resources and developing new food products. Despite the advantages of combining the two, FSG is a weaker emulsifier compared to other surfactants, often resulting in larger droplet sizes when used alone. To enhance its emulsifying properties, FSG is typically modified with non-polar side groups or combined with other surfactants [6]. FSG also shows promise for forming thermoreversible gels in stabilized emulsions, with polysaccharides and mixed polysaccharide systems as potential alternatives [1]. Therefore, the study of well-stabilized food emulsions has gradually become one of the research hotspots in the food discipline. The molecular modification of FSG has also received increasing caution when developing emulsions [7].

Glycosylation modification is the intermolecular binding of polysaccharides to proteins and is an effective method to enhance the emulsification of proteins. The coupling is done directly with hydrophilic polymers through a Maillard reaction. The stability of oil-in-water emulsions can be significantly enhanced by the protein–polysaccharide coupling that is formed [8]. It can happen organically in regulated environments with respect to time, temperature, humidity, and other factors. It is possible to complete it without using any further chemicals [9]. As a result, glycosylation (dry Maillard reaction) is a “green” method of modifying the proteins because its ingredients are edible by nature [10]. Prior research has discovered that the Maillard reaction products exhibit differences from the parent proteins. It has improved solubility, thermal stability, and emulsification qualities [11]. Dex is a neutral and hydrophilic polysaccharide. Alpha-1,6 bonds bind the glucose residues in a linear chain. Its conformation is highly flexible and has good solubility in water. Due to its neutral charge, Dex also inhibits electrostatic complexation between proteins and polysaccharides. This makes dextran an ideal material for reactions with protein glycosylation [12]. The combination of whey isolate protein (WIP) and dextran polysaccharide chains alters the molecular structure and spatial conformation, leading to improved emulsification, solubility, and nanoemulsion stability. While previous studies have demonstrated enhanced functional properties of glycosylated proteins, such as soybean and rice proteins, the underlying improvements have still not been fully explored, especially for FSG. This study aims to address this knowledge gap through the structural modification and functional enhancement of FSG by glycosylation, focusing on its potential as a premium emulsifier in the food system. The main research questions include: how does glycosylation affect the molecular structure of FSG? Whether the solubility and emulsification have been effectively improved [13,14].

This study aims to deepen the understanding of the relationship between the structure of fish skin gelatin (FSG) couplers and their emulsion-stabilizing processes, enhancing the value of FSG for food applications. FSG was glycosylated via the dry Maillard reaction using dextran with molecular weights of 10 kDa, 100 kDa, 200 kDa, and 500 kDa. The degree of grafting (DG) and browning were used to evaluate glycosylation. The microstructures of FSG–Dex glycoconjugates were analyzed using SDS-PAGE, FTIR, fluorescence spectroscopy, and scanning electron microscopy (SEM). Interfacial properties were assessed through emulsifying activity, emulsifying stability, hydrophobicity index, liquid potentiometry, particle size, polydispersity index (PDI), and zeta potential of emulsions. Confocal laser scanning was used for further analysis, while DPPH and ABTS radical scavenging activities were measured to evaluate antioxidant properties. A comprehensive examination of the connection between structural modifications in FSG–Dex conjugates and their emulsification and antioxidant characteristics was conducted, which may identify mechanisms to improve FSG’s emulsifying performance.

## 2. Materials and Methods

### 2.1. Materials

FSG was purchased from Shanghai Yuan Ye Technology Co., Ltd. (Shanghai, China). Dex was purchased from Shanghai Macklin Biochemical Co., Ltd. (Shanghai, China). Litsea cubeba oil was purchased from Chongqing Tree Fresh Food Co. (Chongqing, China). Shanghai Rhawn Chemical Technology Co., Ltd. (Shanghai, China) was the supplier of mixed amino acid standard solution, o-phthalaldehyde (OPA), potassium hydroxide, 8-Anilino-1-naphthalenesulfonic acid (ANS), and other reagents. Every additional chemical utilized in this investigation was of analytical quality.

### 2.2. Preparation of FSG–Dex Couplings

The preparation method was slightly modified according to Hong et al. [15]. FSG was dissolved in water and stirred in a thermostatic bath at 50 °C until fully dissolved at a ratio of 1:1. Subsequently, dextran (10 kDa, 100 kDa, 200 kDa, 500 kDa) was added. The sample solution was prepared in deionized water at 6% (*w*/*v*) and underwent freeze–drying to form a powdered state. Within a constant temperature and humidity chamber (model JK-40–225, Shanghai Jiangkai Instrument, Shanghai, China), the relative humidity was maintained at 79%, while the temperature was held at 60 °C for 1 week to facilitate the formation of gelatin–dextran coupling. The gelatin–dextran couplers were designated as FSG–Dex10, FSG–Dex100, FSG–Dex200, and FSG–Dex500, while their corresponding physical mixtures were denoted as Mix10, Mix100, Mix200, and Mix500.

### 2.3. Browning and Degree of Grafting

Dissolving gelatin–dextran couplers and physical mixtures (5 mg/mL) in water. Absorbance measurements were performed using a UV-2500 spectrophotometer (T9CS, Beijing Persee General Instrument, Beijing, China) at 420 nm, with water serving as the blank.

The measurement method of the degree of grafting (DG) of gelatin–dextran couplers was slightly modified according to Liu et al. [16]. The o-phthalaldehyde (OPA) was prepared and measured. The absorbance of these samples was measured at 340 nm using a UV-Vis spectrophotometer. DG was calculated as follows:

DG(%) = *C*_0_ − *C_t_/C* × 100% (1)

where *C*, *C*_0_, and *C_t_* are the free amino group molar concentrations in FSG, FSG–dextran couplers, and FSG–dextran mixes.

### 2.4. Sodium Dodecyl Sulfate–Polyacrylamide Gel Electrophoresis (SDS-PAGE)

SDS-PAGE was performed in 0.5 mol/L Tris-HCl buffer (pH 6.8), in which concentrations of the stacking gel and separating gel were 5% and 6%, respectively. The sample was carried out in an electrode buffer containing 25 mmol/L Tris, 192 mmol/L glycine, and 0.1% SDS, pH 8.3. A volume of 10 μL of the protein sample (10 mg/mL) was loaded onto the gel. After electrophoresis, the gel was stained with Coomassie Brilliant Blue for 40 min and then decolorized with a decolorizing solution (methanol/glacial acetic acid/water, 1: 1: 8, *v*/*v*) to clear. Standard protein markers ranging from 25 to 400 kDa were included. Subsequently, it was destained using a decolorizing solution consisting of methanol. The electrophoresis instrument voltage was operated at a voltage of 120 V.

### 2.5. FT-IR Analysis

The sample was mixed with potassium bromide pellets at a mass ratio of 1:100. Utilizing an FTIR spectrometer (BRUKER VERTEX 70, Ettlingen, Germany) with a wave number precision of 0.01 cm^−1^ and scanning frequency in the range 400–4000 cm^−1^. Samples were analyzed with SeaSolve PeakFit software (San Jose, CA, USA, Version 4.12) to determine the proportion of secondary structure in the monosaccharide-modified gelatin.

### 2.6. Fluorescence Analysis of the FSG–Dex Glycoconjugates

Sample solutions with a consistent protein concentration of 1 mg/mL were prepared using 10 mmol/L phosphate buffer (pH 7.0). The fluorescence emission spectra were recorded using a fluorescence spectrophotometer (FLS1000, Edinburgh Instruments, Livingston, UK). The excitation wavelength was set at 280 nm. Emission wavelengths from 300 to 500 nm were scanned during the experiment.

### 2.7. Measurement of Surface Hydrophobicity

The ANS fluorescent probe method was employed to assess the protein’s hydrophobicity [17]. Protein samples were dissolved in 0.01 mol/L phosphate buffer. Fluorescence intensity measurements were performed with the excitation wavelength set at *λ_ex_* = 390 nm and emission wavelength set at *λ_em_* = 470 nm.

### 2.8. Emulsion Activity (EAI) and Emulsion Stability (ESI)

Several refinements were implemented based on Zhang et al.’s previous methodology [2]. The control gelatin and glycosylation products were dissolved in distilled water (1%, *w*/*w*). Following emulsification, 6 mL of protein solution and 2 mL of Litsea cubeba oil were combined to form the final emulsion. A high-speed homogenizer (AD500S-H, Angni instruments, Shanghai, China) operating at 15,000 rpm for one minute was used to process the samples. Emulsion samples were then diluted fifty-fold (60 μL) with SDS solution, and absorbance was measured at 500 nm. The equations for calculating the emulsion activity index (EAI) and emulsion stability index (ESI) equations were as follows:

EAI (m^2^/g) = 2 × 2.303 × *A*_0_ × *DF*/*C* × *ø* × (1 − *θ*) × 10,000 (2)

ESI (min) = *A*_0_/(*A*_0_ − *A_T_*) × *T* (3)

where *A*_0_ and *A_T_* are the emulsion’s absorbance at 500 nm after 0 min and *T* min, *C* is the protein content (4.81 g/mL), *DF* is the dilution factor (*DF* = 50), *ø* is the optical path (*ø* = 0.01 m), and *θ* is the amount of oil phase in the emulsion (*θ* = 0.25).

### 2.9. Scanning Electron Microscopy (SEM) Analysis

The sample is freeze–dried to form a powder, which is then placed on a conductive adhesive and then sputtered for gold-plating. Images were taken using an S-4800 scanning electron microscope (Apreo 2S, Thermo Fisher Scientific, Waltham, MA, USA) with an accelerating voltage of 20 kV and an instrument magnification of 400×.

### 2.10. Liquid Potentiometry

The particle size and potential of the nanoemulsions were measured by means of a Malvern particle sizer (Nano-ZS90, Malvern Instruments, Malvern, UK). Prior to testing the samples, they were diluted with gelatin dextran conjugate 50-fold.

### 2.11. Particle Size, PDI, and Zeta Potential of Emulsions

The particle size and potential of the nanoemulsions were measured by means of a Malvern particle sizer (Nano-ZS90, Malvern Instruments, Malvern, UK). Prior to testing the samples, the original emulsion was diluted 100-fold.

### 2.12. Laser Scanning Confocal Microscope

Freshly prepared Nile Blue staining solution (20 µL, 0.2%, *w*/*v*) was added to 1 mL of emulsion. Proteins were allowed to stain evenly, and the morphology of the emulsion was observed by CLSM (A1RHD25, Nikon, Tokyo, Japan) [18].

### 2.13. DPPH Radical Scavenging Activity

The emulsion was tested for DPPH free radical scavenging according to the method described by Yu et al., and slight modifications were made [19]. Preparations of FSG–dextran coupler emulsions were created with different concentrations (0.1–1.0 mg/mL). The 1 mL sample was simply mixed with 3 mL DPPH ethanol solution (0.2 mmol/L). The sample was incubated at 37 °C for 30 min, and the absorbance of the sample (As) was measured at 517 nm using a multifunctional UV microporous plate instrument (EPOCG2, Bio Tek, Winooski, VT, USA). A blank sample (Ab) was obtained by replacing DPPH in the emulsion sample with ethanol. The control sample (Ac) was replaced by a mixture of distilled water and DPPH solution. DPPH clearance is calculated as follows:

DPPH scavenging rate % = [1 − (A_s_ − A_c_)/A_b_ ] × 100 (4)

### 2.14. ABTS Radical Scavenging Activity

In this assay, 0.2 mL of ABTS (7.4 mmol/L) was mixed with 0.2 mL of K_2_S_2_O_8_ (2.6 mmol/L) and left undisturbed for 12 h at room temperature in the dark. Subsequently, the ABTS working solution was prepared by dilution until the absorbance value reached 0.70 ± 0.02. For the assay, 0.8 mL of the ABTS working solution was mixed with 0.2 mL of the sample under investigation. The absorbance (A) of the mixture was recorded after a 6 min incubation at 734 nm. A control sample (A_0_) was obtained by substituting the sample with 0.2 mL of 95% ethanol. The ABTS scavenging rate was calculated using the following formula:

ABTS scavenging rate % = [A_0_ − (A_0_ − A)/A_0_] × 100 (5)

### 2.15. Statistical Analysis

All experiments were conducted in triplicates. The obtained results were presented as mean ± standard deviation. Statistical analysis of the data was performed using statistical analysis software (SPASS 25.0, SPSS Inc., Chicago, IL, USA). Significant differences between the means of the samples were determined using a one-way ANOVA test (Duncan’s test), and post-hoc analysis was carried out using the Duncan’s test. A significance level of *p* < 0.05 was considered for establishing statistical differences.

## 3. Results and Discussion

### 3.1. Degree of Browning and Degree of Grafting

The extent of glycosylation of the protein during the Maillard reaction is directly indicated by the resulting degree of DG. Initially, sugars and protein combine to form amino condensation, followed by Maillard rearrangement, leading to the formation of Schiff Bases. This process yields high molecular glycosylation products, accompanied by the development of browning compounds. Consequently, the abundance of free amino groups diminishes as the reaction progresses. DG serves as a measure by calculating the content of free amino products in the reaction groups [20]. Additionally, browning is another crucial parameter for evaluating the intensity of glycosylation reactions [21].

**Figure 1:**
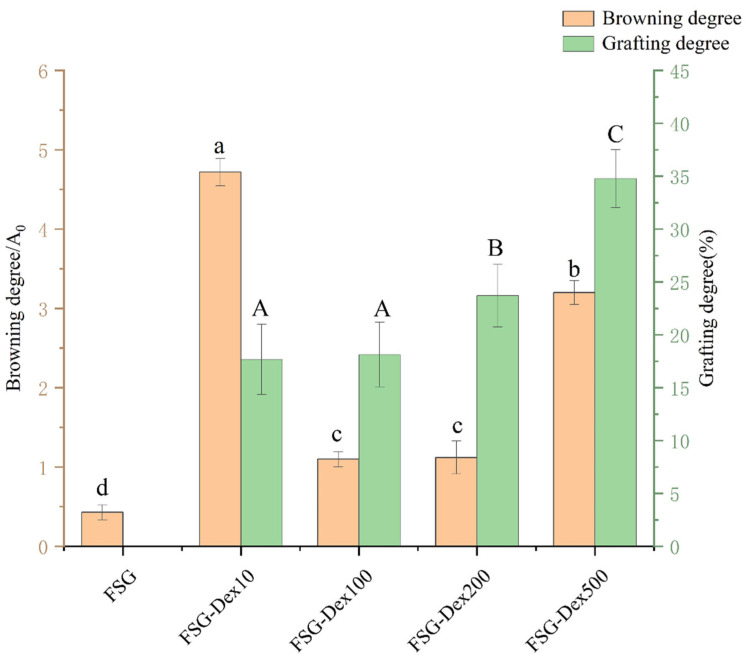
Changes in grafting degree and final product (A420) of FG-Dex affixes. FSG: untreated fish skin gelatin; FSG–Dex 10, FSG–Dex 100, FSG–Dex 200, FSG–Dex 500: fish skin gelatin modified with different molecular weight dextran glycosylation (10 kDa, 100 kDa, 200 kDa, 500 kDa). Different lowercase letters indicate significant differences in grafting degree among different samples (*p* < 0.05), and different capital letters indicate significant differences in browning degree among different samples (*p* < 0.05).

Figure 1 demonstrates the relationship between DG and the molecular weight of Dex, showing an increase in DG as the molecular weight of Dex rose. The highest DG value was observed at a molecular weight of 500 kDa (34.77%), followed by 200 kDa (23.70%), 100 kDa (18.13%), and 10 kDa (17.67%). These results suggest that glycosylation processes enhanced protein–polysaccharide interactions, likely due to the promotion of coupling formation in a crowded macromolecular environment. It is plausible that the increased molecular weight of Dex alters the spatial conformation of FSG glycosylated proteins, possibly facilitated by the interaction of the early reaction of free amino groups on the protein surface with Dex. This results in the generation of highly glycosylated products, thereby increasing DG. Among the different molecular weight groups of dextran, pronounced browning was observed in Dex with a molecular weight of 10 kDa, likely due to its smaller size, which enhances reactivity and reduces spatial site resistance for bound polysaccharide molecules, thereby limiting the binding rate [22]. In comparison to low molecular weight dextran, high molecular weight dextran exhibits a greater mass fraction of carbonyl groups and spatial resistance. Consequently, the pathway for carbonyl groups to bind to the protein’s amino group is reduced, as suggested by studies [23].

### 3.2. SDS-PAGE Analysis

SDS-PAGE was used to evaluate the formation of FSG–Dex, and the results showed that the electrophoresis bands and mobility of the FSG–Dex mix are comparable to those of FSG alone. Graphs illustrated negligible variations in composition or molecular weight compared to mixtures from FSG. Without reduction, characteristic protein molecular weight bands of FSG and its Dex mixture are primarily distributed within ranges of 75–80 kDa, 120–130 kDa, 150–160 kDa, and 245 kDa. Covalent bonds are formed between the amino acids of the protein and the reduced end of the sugar as a result of their interaction. This is because glycosylation leads to an increase in the molecular weight of FSG–dextran complexes. Glycosylated proteins exhibit larger molecular weights, causing them to migrate near the top of the gel [24].

**Figure 2:**
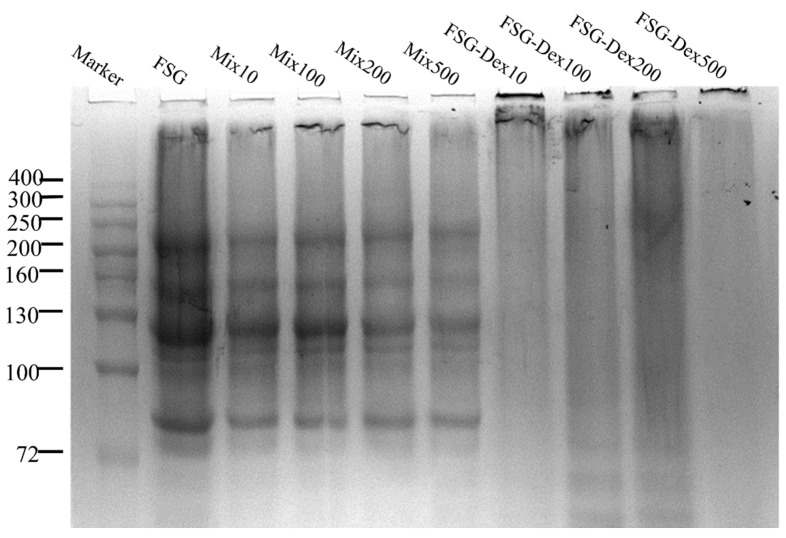
FSG–Dex conjugate after reaction, FSG–Dex mixture prior to reaction, and FSG SDS-PAGE of all three.

As can be observed in Figure 2, compared with the FSG–Dex mixture, the protein molecular weight bands of the glycosylation product largely diminished, showing a tendency to lighten. Additionally, bands with larger molecular weights were observed near the top of the separating gel. Bands in the FSG–Dex lanes were all shifted upward or lightened compared with the mixture and control FSG lanes, consistent with previous findings [25]. These findings support the development of covalent combinations with higher molecular weights resulting from the glycosylation of FSG and Dex, suggesting that Dex binds to FSG during this process. This leads to an increase in large-molecule couplers and a decrease in FSG components. The FSG–Dex complex did not exhibit dense bands in the swim lane, suggesting the creation of a complex mixture.

### 3.3. Structural Characterization of FSG–Dex Conjugates 

#### 3.3.1. Fourier Transform Infrared (FTIR) Analysis

**Figure 3:**
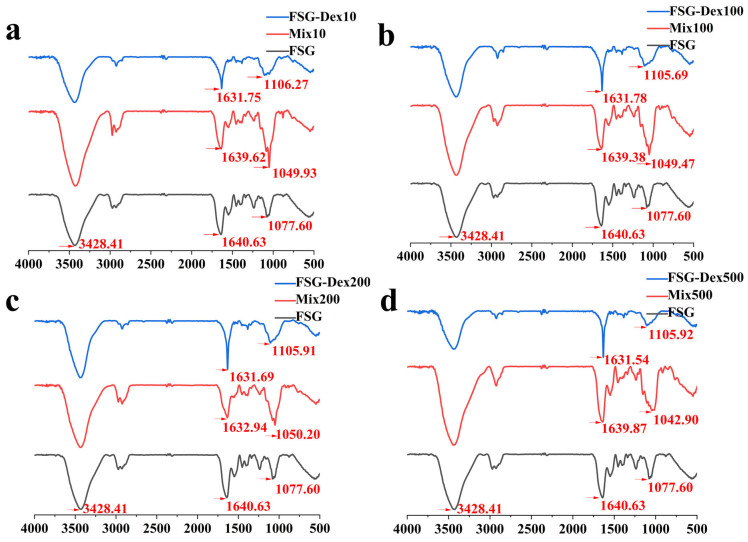
FITR of FSG, FSG−Dex mixtures, and FSG−Dex conjugates (**a**–**d**).

FTIR spectroscopy serves as a valuable tool for probing the secondary structure of proteins, allowing for the detection of protein structural changes. According to Figure 3, a broad peak was observed in the FSG–Dex conjugates within the range of 3200–3700 cm^−1^, attributed to the stretching vibration of free OH groups. This peak exhibited a broader apex compared with the original FSG spectrum, indicating that strong intramolecular hydrogen connections are created when a significant amount of dextran is grafted onto the FSG via covalent bonding [26]. Additionally, the absorption peak of the conjugates was higher than that of natural FSG in the range of 950–1200 cm^−1^, signifying the alteration of FSG’s internal structure and the successful grafting of polysaccharides onto the protein [27]. The characteristic absorbance peak of Amide A arises from the stretching vibrations of NH bands. Reduction in the frequency of amide A’s absorption peak indicates the formation of hydrogen bonds by NH groups, suggesting their involvement in hydrogen bonding in glycosylated gelatin [28].

**Figure 4:**
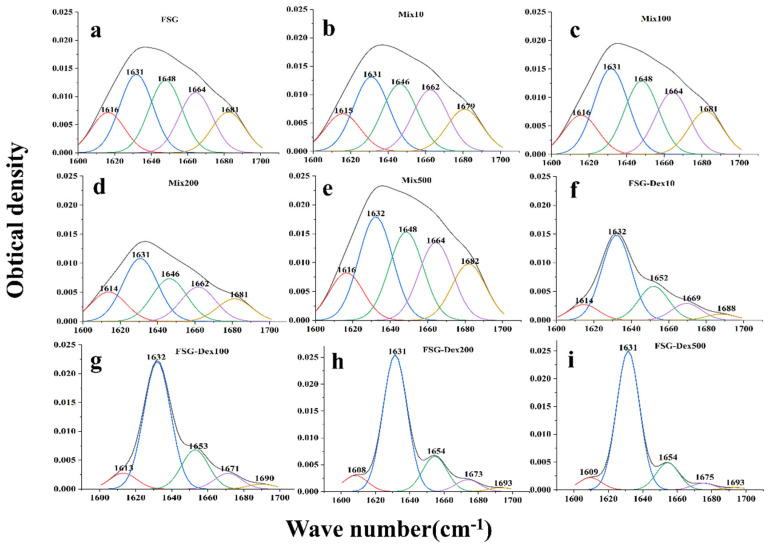
Deconvolution infrared spectra of amide I bands of untreated FSG, FSG–Dex mixtures, and glycosylated FSG–Dex conjugates samples. (**a**) Original FSG sample. (**b**–**e**) Mix10, Mix100, Mix1200, Mix500. (**f**–**i**) FSG−Dex10, FSG−Dex100, FSG−Dex200, FSG−Dex500. Black line: Original FTIR spectrum showing overall absorbance. Colored lines (red, blue, green, purple, orange): Deconvoluted peaks of the amide I region, indicating protein secondary structures. Red: β-sheets (~1610–1630 cm^−1^). Blue: α-helices (~1648−1660 cm^−1^). Green: Random coils (~1640–1648 cm^−1^). Purple/Orange: β-turns (~1662–1680 cm^−1^).

According to Figure 4, based on an area of 1600–1700 cm^–1^, the secondary structure of gelatin was examined [29]. Each secondary structural unit’s relative composition was determined, and Table 1 illustrates this. Compared with the control gelatin, the secondary structure of gelatin with added dextran before the reaction remained essentially unchanged. However, after the dry Maillard reaction, the dextran–gelatin conjugates exhibited an increase in β-folding, accompanied by the disappearance of irregular curls, an increase in α-helices, and a decrease in β-turns and β-antiparallel content. Ordered structures consist of α-helical and β-folded structures, while disordered structures include β-turns and irregularly curled structures [30]. This suggested that the dry Maillard process facilitates the transition of proteins from an ordered to a disordered structure. During the initial stage of dry Maillard treatment, proteins likely unfold and denature, potentially leading to rebinding or aggregation due to hydrophobic or other interactions. Subsequent stages of the dry Maillard treatment or post-reaction release may then involve the restructuring of damaged secondary structures [31].

This behavior can be attributed to the binding of unfolded or denatured proteins through hydrophobic and other weak interactions [32]. Additionally, heating reactions strengthen hydrophobic interactions, promoting aggregation, consistent with the later results regarding enhanced hydrophobicity of gelatin surfaces [33]. Elevated β-sheets and a stabilized triple-helical conformation are associated with gelatin possessing a dense network structure, while reacted FSG exhibits distinct qualities, including lipophilicity and hydrophilicity [34]. As β-sheets swell, gelatin becomes more hydrophilic, contributing to a more uniform network structure [35].

#### 3.3.2. Intrinsic Fluorescence Analysis

Intrinsic fluorescence spectroscopy, measuring wavelengths between 300 and 500 nm, is utilized to assess modifications in protein molecules, particularly those close to tertiary structures and hydrophobic residues in microenvironments [36]. This spectroscopic technique proves effective in analyzing protein spatial structures, especially considering that glycoconjugates are spontaneous products, making fluorescence spectroscopy a valuable tool for studying their structure [37].

**Figure 5:**
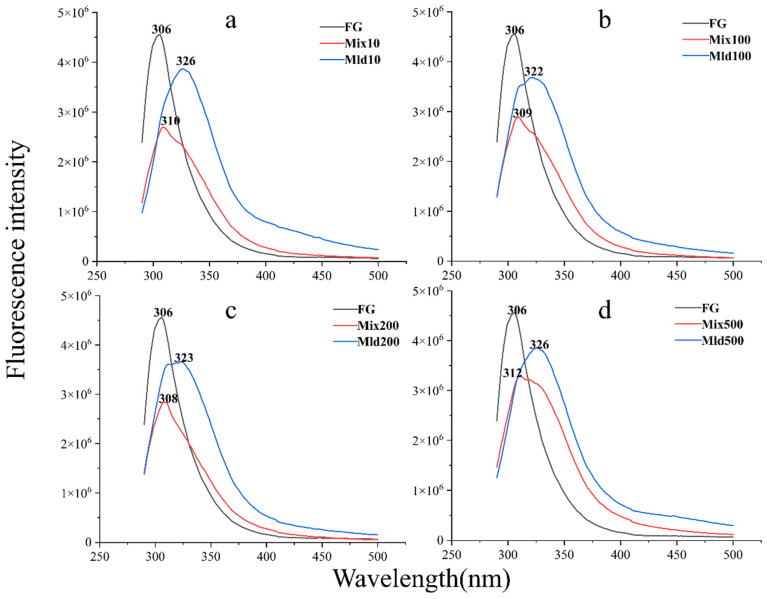
Intrinsic fluorescence of FSG, FSG–Dex mixtures, and FSG–Dex conjugates (**a**–**d**).

Figure 5 depicts the intrinsic fluorescence spectra of natural FSG, FSG–Dex mixtures, and FSG–Dex conjugates. At an excitation wavelength of 280 nm, untreated FSG exhibited the highest absorption wavelength (*λ_max_*) at 306 nm. However, the intensity of the FSG–Dex mixture decreased compared to the *λ_max_* of untreated FSG, likely due to polysaccharide chains blocking specific spatial sites [38]. This trend is evident when compared to FSG. As sugar molecules infiltrated the internal structure of the conjugates, their fluorescence spectral absorbance gradually diminished, stabilizing with a red shift in *λ_max_*. The maximum absorptions of the conjugates (10 kDa, 100 kDa, 200 kDa, and 500 kDa) were red-shifted to 326 nm, 322 nm, 323 nm, and 326 nm, respectively. This phenomenon can be attributed to hydrophobic groups within the FSG molecule encountering the protein’s surface and relocating to a more hydrophilic environment during the glycosylation process [39]. As more dextran is absorbed by the protein, a portion of the fluorescence emission intensity is obscured, leading to reduced fluorescence absorption compared to FSG. Moreover, the tertiary structure of FSG becomes more relaxed, allowing for increased doping of Dex into the FSG and enhancing the local polar environment. Similar studies have underscored the correlation between the structure of glycoconjugates and fluorescence intensity, indicating the impact of covalent grafting alterations on the protein fluorophore’s surrounding milieu, consistent with existing literature [40].

#### 3.3.3. Scanning Electron Microscopy (SEM)

**Figure 6:**
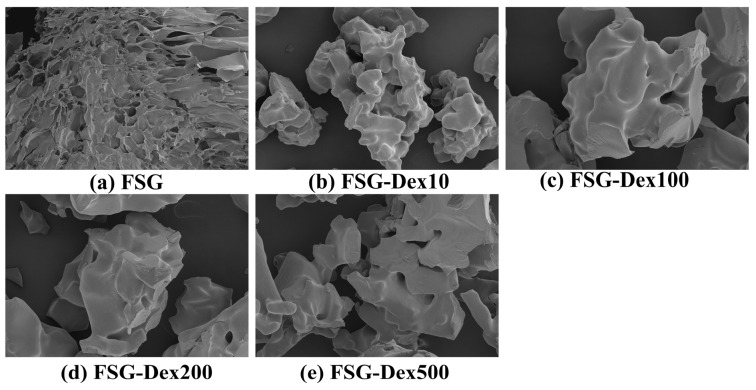
(**a**) Fish skin gelatin and (**b**–**e**) conjugate with different molecular weights.

The SEM images (Figure 6) illustrated significant structural changes in the FSG matrix after the addition of dextran (Dex) with varying molecular weights. Compared with FSG, the conjugates exhibit noticeable changes in their microstructure, indicating that the dry Maillard reaction and glycosylation modified the FSG conformation. The untreated FSG displays a dense, porous lamellar structure with a rough surface and loose texture. In contrast, the processed conjugates take on a spherical shape, forming smooth aggregates. The original FSG exhibits a porous, layered structure, while the incorporation of low molecular weight Dex (Dex10) leads to a more compact and granular morphology. As the molecular weight increases from Dex100 to Dex200 and Dex500, the samples gradually display irregular, porous, and sheet-like surface features, becoming more loosely structured. These changes suggested that the increasing molecular weight of Dex enhanced internal interactions within the material, forming more complex networks and layered structures, potentially affecting properties such as surface area, adsorption capacity, and other physicochemical characteristics. This transformation was likely due to the glycosylation process, which compacted the protein fibers. Furthermore, the pore size was reduced, and the edges became smoother [41]. The functionality of the protein was improved, facilitating the aggregation of polysaccharides on the protein surface.

### 3.4. Features of FSG–Dex Conjugate Functionality

#### 3.4.1. Hydrophobicity Analysis

The surface hypothesis index (H_0_) suggests that interactions occur between the hydrophobic groups of the protein and its surroundings, significantly influencing the protein’s emulsification capacity [42]. An essential indicator of the emulsification process is the surface hydrophobicity (H_0_) of FSG, which are amphiphilic proteins with high surface hydrophobicity, facilitating their migration from the aqueous phase to the interface between oil and water. This migration occurs due to hydrophobic interactions between the non-polar parts of the FSG polypeptide chain, resulting in the formation of a layer that stabilizes oil droplets in the emulsion [43].

**Figure 7:**
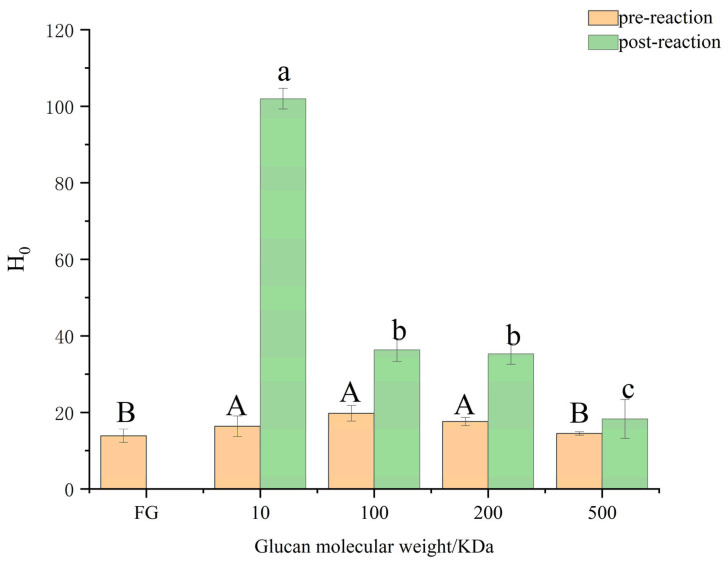
Surface hydrophobicity index (FG: fish skin gelatin;10, 100, 200, 500: represent the corresponding molecular weight of dextran, respectively). Different lowercase letters indicate significant differences in post-reaction among different samples (*p* < 0.05), and different capital letters indicate significant differences in pre-reaction among different samples (*p* < 0.05).

Figure 7 illustrates that FSG–Dex conjugates, after the Maillard reaction, exhibit significantly higher surface hydrophobicity than FSG alone. The molecular weight of dextran increased by 553.78% for 10 kDa, 83.89% for 100 kDa, 100.56% for 200 kDa, and 26.15% for 500 kDa. This increase may have resulted from aggregate dissociation or protein unfolding. The Maillard process induced protein denaturation, altering the protein’s internal molecular structure. As the molecular weight of the additional dextran increased, the surface hydrophobicity of the FSG conjugates decreased, possibly due to the reduction in hydrophobicity with the increasing glycosylation level [44].

Protein denaturation occurs upon heating, exposing naturally present hydrophobic groups within the molecule, leading to an increase in surface hydrophobicity [45]. Feng et al. also observed this phenomenon [44]. Elevated temperatures promote the exposure of native hydrophobic groups within FSG molecules. The surface hydrophobicity of sugar-conjugated complexes decreases gradually as the molecular weight increases from 10 kDa to 500 kDa. This reduction occurs due to structural alterations in proteins upon the introduction of polyhydroxy sugar molecules, enhancing intermolecular hydrophilicity. Consequently, the overall surface hydrophobicity of glycoalkaloids decreases. Thus, it is hypothesized that the degree of grafting increases with the molecular weight of dextran, as evidenced by the data in Section 3.1. The incorporation of dextran chains may shield the hydrophobic components of FSG, resulting in a relative decline in total surface hydrophobicity of FSG–Dex conjugates [46].

In contrast, the Maillard reaction typically impacts protein structure by partially disrupting the two and three-tiered structures of FSG. This reaction led to the depolymerization of the polypeptide chain, exposing hydrophobic sites without significant aggregate formation, consequently increasing surface hydrophobicity [47]. Sun et al.’s study suggested that the Maillard reaction can unfold the internal structure of proteins, potentially exposing tryptophan groups previously encapsulated within the protein structure, resulting in increased surface hydrophobicity. Longo et al. [48] observed that glycosylation modification enhances the binding of hydrophilic molecules (glycosidic chains) to proteins, thereby promoting quicker elution in reverse-phase HPLC. Stronger modifications exert a more pronounced influence on the balance between protein surface hydrophobicity and hydrophilicity.

#### 3.4.2. Zeta Potential, Particle Size, and PDI

**Figure 8:**
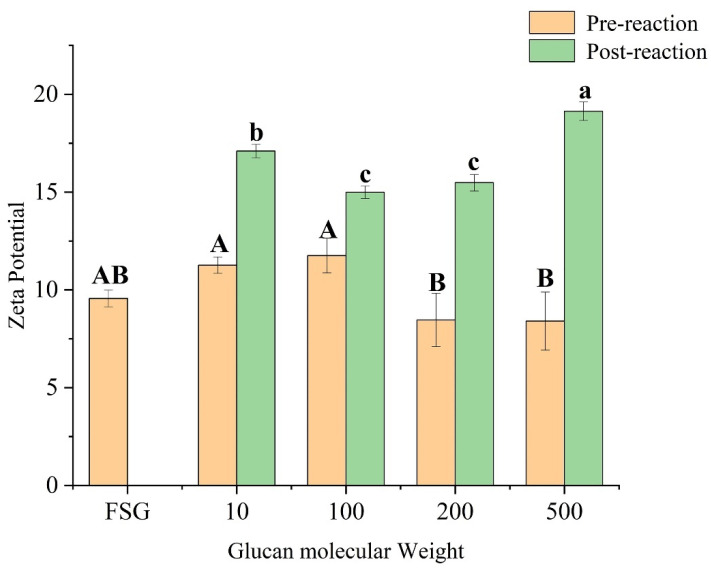
Conjugate liquid potential (FSG: fish skin gelatin;10, 100, 200, 500: represent the corresponding molecular weight of dextran, respectively). Different lowercase letters indicate significant differences in post-reaction among different samples (*p* < 0.05), and different capital letters indicate significant differences in pre-reaction among different samples (*p* < 0.05).

Zeta potential is a critical indicator of the stability of colloidal dispersion systems. Smaller molecules or dispersed particles tend to have higher absolute values of zeta potential (positive or negative), which increases the repulsive forces between particles and enhances system stability. Surface charge is a key factor that significantly affects protein stability [49]. As shown in Figure 8, glycosylation affected the surface charge of FSG, resulting in a significant increase in zeta potential. This may be due to the glycosylation process increasing the net negative charge of the protein. While dry Maillard accompanies thermal reactions, heating may also play a role. During heating, charged amino acids may become embedded in the molecular structure, leading to a significant increase in the absolute value of the zeta potential. Compared with the untreated mixture, the dry Maillard reaction and glycosylation allow FSG to expose more positively charged amino acids to the surrounding environment [50].

**Figure 9:**
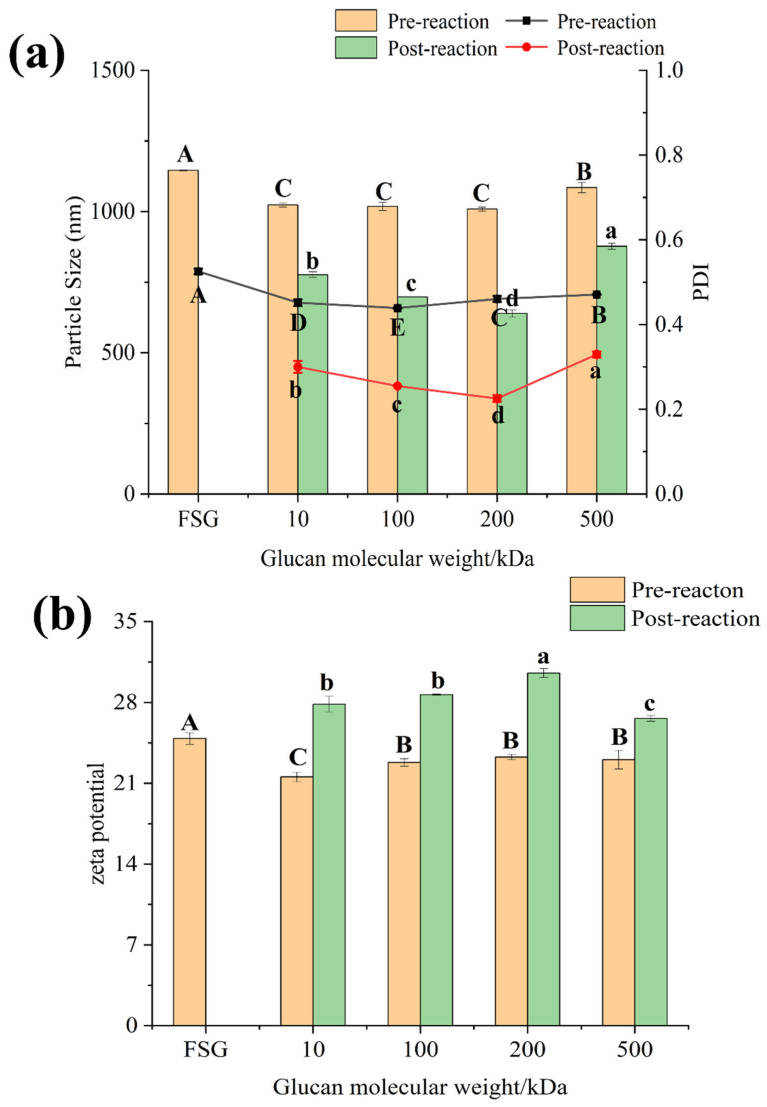
(**a**) Particle size of the emulsion and PDI and (**b**) potential (FSG: fish skin gelatin; 10, 100, 200, 500: represent the corresponding molecular weight of dextran, respectively). Different lowercase letters indicate significant differences in pre-reaction among different samples (*p* < 0.05), and different capital letters indicate significant differences in post-reaction among different samples (*p* < 0.05).

Figure 9a presents the particle size, polydispersity index (PDI), and zeta potential of FSG and the glycosylated conjugates. The results indicated a significant reduction in particle size for the conjugates compared with the mixture. The particle size of the mixture and FSG emulsions ranged from 1009 to 1146 nm, while the glycosylated conjugate emulsions ranged from 770 to 877 nm. Specifically, the FSG–Dex 200 kDa conjugate had the smallest particle size at 639.1 nm, and the FSG–Dex 500 kDa conjugate had the largest at 877.35 nm. This suggested that the glycosylated conjugates exhibit strong emulsification ability and stability. This reduction in droplet size may be attributed to the disruption of the protein’s original globular structure by polysaccharides after covalent bonding. Additionally, the increased surface charge of the conjugates post-glycosylation, along with enhanced electrostatic interactions between the proteins and polysaccharides, could further contribute to the reduction in droplet size [51]. The PDI value of FSG is the highest (0.52 ± 0.07), indicating that fish skin gelatin protein molecules tend to aggregate and form larger particles. After glycosylation treatment, both the particle size and PDI of the conjugates significantly decreased. This reduction is likely due to the protective effect of sugars against protein denaturation during heat treatment, which minimizes protein molecule aggregation [52].

As shown in Figure 9b, the absolute value of the zeta potential for the mixture before the reaction is relatively low compared to the conjugates, indicating weaker electrostatic repulsion. The absolute zeta potential values of the conjugates are all close to 30 mV, suggesting that all samples belong to a stable system. After glycosylation modification, the absolute value of the zeta potential increases due to the rise in net negative charge. This indicated that the conjugates possess a higher charge density [53]. Glycosylated covalent conjugates exhibit higher charge density compared to non-covalent conjugates. This is primarily attributed to the stronger binding forces, greater number of binding sites, and more compact interactions present in covalent conjugates [54]. In summary, glycosylation reactions can produce emulsions with smaller droplet sizes, lower PDI values, higher zeta potential, and enhanced stability.

#### 3.4.3. Laser Scanning Confocal Microscope

**Figure 10:**
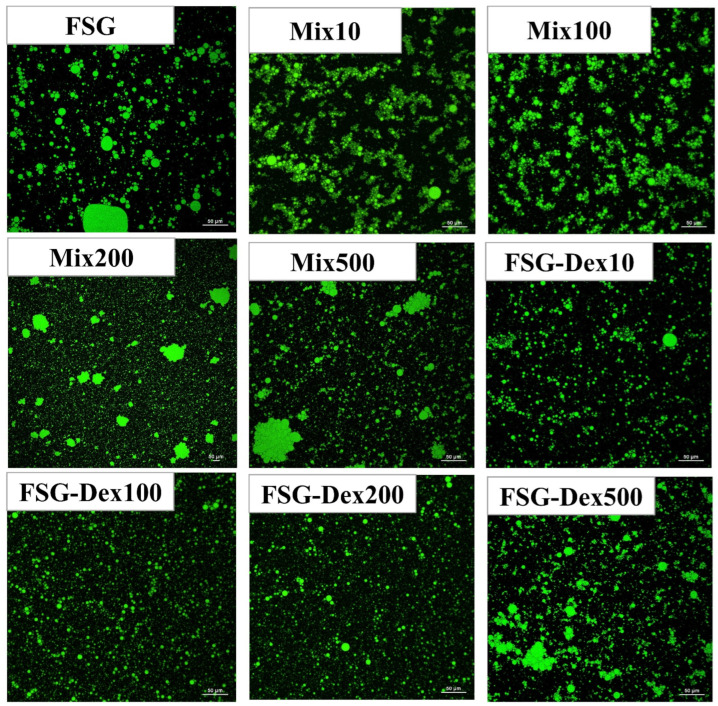
CLSM (FSG: fish skin gelatin; Mix10, Mix10, Mix100, Mix200, Mix500: mixtures of different molecular weights; FSG–Dex10, FSG–Dex100, FSG–Dex200, FSG–Dex10, FSG–Dex100, FSG–Dex200, FSG–Dex500: couplings of different molecular weights.).

In the laser confocal scanning images shown in Figure 10, the bright regions represent protein droplets. The CLSM of the emulsions stabilized by FSG and its glucan mixture indicates that the droplets exhibit rapid coalescence behavior, resulting in larger droplets. In contrast, the emulsions stabilized by the conjugates demonstrated slower droplet coalescence. This difference can be attributed to the changes in protein conformation caused by the incorporation of sugars during the dry Maillard reaction, which disrupts aggregation between proteins. The presence of sugars introduces steric hindrance, affecting interactions between the proteins. As the sugar content increased, the protein aggregates gradually broke down more significantly [55]. The aggregated gradually decreased, reaching a peak at a molecular weight of 200 kDa. As the molecular weight of the glucan increased, there was a subsequent rise in the aggregates. On the other hand, the increased surface charge of the conjugates enhances the electrostatic interactions between droplets, leading to greater repulsion. As a result, the droplets are less likely to coalesce [56]. The results indicated that after glycosylation and covalent bonding, the balance between the hydrophilic and hydrophobic properties of the conjugate surfaces becomes more coordinated, leading to an increase in surface charge. Consequently, these conjugates exhibited optimal emulsification and stability. The glycosylation reaction exerted an inhibitory effect on the coalescence behavior of emulsion droplets, allowing the emulsions to maintain good stability and indirectly demonstrating an increase in emulsification efficiency [57].

#### 3.4.4. Emulsification Performance Analysis

**Figure 11:**
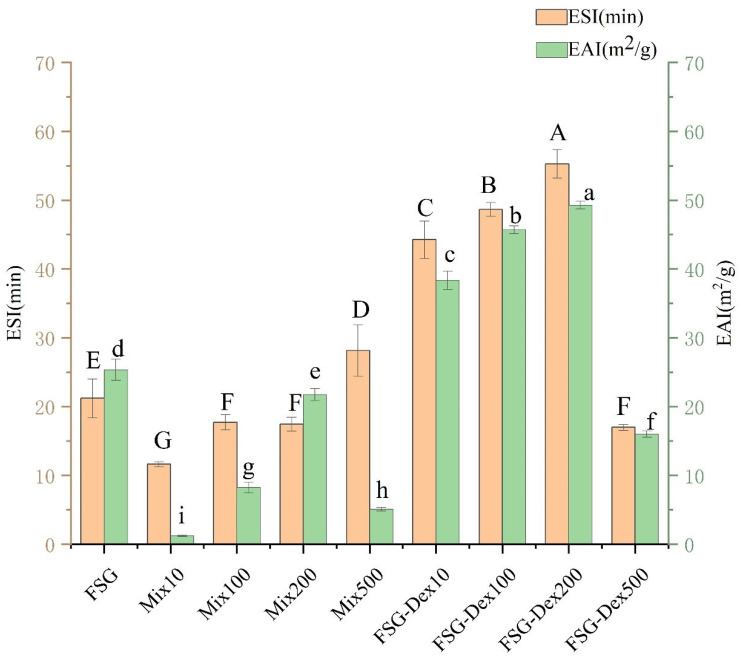
EAI and ESI (FSG: fish skin gelatin; Mix10, Mix10, Mix100, Mix200, Mix500: mixtures of different molecular weights; FSG–Dex10, FSG–Dex100, FSG–Dex200, FSG–Dex10, FSG–Dex100, FSG–Dex200, FSG–Dex500: couplings of different molecular weights). Different lowercase letters indicate significant differences in EAI among different samples (*p* < 0.05), and different capital letters indicate significant differences in ESI among different samples (*p* < 0.05).

Due to proteins’ strong affinity for the oil/water interface, the development of protein–polysaccharide glycoconjugates is highly encouraged. The spatial distribution of polysaccharides in the aqueous phase also plays a contributing role [58], imparting favorable emulsification behavior to these conjugates. This behavior hinges on the interplay between molecular flexibility, amphiphilicity, and conformation. Two common metrics used to evaluate emulsification capabilities are ESI and EAI [59]. Figure 11 depicts changes in emulsification characteristics before and after the FSG grafting reaction. Compared to natural FSG, the emulsification activity of FSG–Dex10, FSG–Dex 100, and FSG–Dex 200 increased by 51.40%, 80.19%, and 94.39%, respectively. Emulsification stability also rose by 108.67%, 129.32%, and 160.49%, respectively. This indicated that with a higher relative molecular weight of dextran, the enhancement of emulsification performance through coupling compounds becomes more pronounced.

Proteins can encircle the surface of oil droplets to form an interfacial layer in O/W emulsions, preventing emulsion separation and droplet aggregation [60]. A higher degree of glycofixation exposes more hydrophobic and hydrophilic groups, thereby increasing the surface area of glycofix molecules. This enhances the hydrophilic impact of the polysaccharide fraction and the protein’s ability to bind at the interface. Covalent graft modification with macromolecular dextran increased intermolecular resistance, leading to stronger adherence between conjugates at the oil–water interface and a thicker protective layer. Moreover, the unfolding of FSG molecules exposes more hydrophobic groups, enhancing the protein’s surface mobility and thus improving emulsification characteristics [61]. Both the conjugated particle size and interfacial resistance increase with the molecular weight of dextran, resulting in a thicker interfacial coating and enhanced emulsification properties with dextran molecular weights of 10, 100, and 200 kDa [62]. Comparisons with low molecular weight dextran aid in understanding this phenomenon, where the high viscosity of high molecular weight dextran prevents oil droplets from floating.

The improved emulsification of FSG–dextran conjugates is clearly demonstrated through multiple analyses, with dextran molecular weight playing some role. Dextran, being hydrophilic, modifies FSG in two ways: it increases the hydrophilicity of the conjugate and decreases surface hydrophobicity, thus reducing the diffusion rate. Additionally, dextran increases the molecular weight of FSG–dextran conjugates, enhancing adsorption capacity. As shown in SEM images, as dextran molecular weight increased, FSG transformed from a porous, lamellar structure into smoother, spherical aggregates, promoting better surface interaction and adsorption capacity. Zeta potential analysis revealed a rise in surface charge post-glycosylation, with higher dextran molecular weights leading to increased electrostatic repulsion and improved system stability. The relationship between these factors and dextran molecular weight was apparent; for instance, dextran glycosylation enhanced protein flexibility, encouraging rearrangement and adsorption at the oil-water interface. The hydrophilic and hydrophobic chain segments of the coupling were balanced when the molecular weight of dextran (200 kDa) was marginally less than that of FSG, resulting in optimal surfactant characteristics. Conversely, excessive hydrophilicity occurs when the molecular weight of dextran (500 kDa) exceeds that of FSG, hampering surface activity, as supported by relevant literature [63]. Smaller particle sizes and lower PDI values, particularly for higher molecular weight dextran conjugates like Dex200, highlight stronger emulsification. For instance, emulsification efficiency was higher with dextran molecular weights (5, 10, and 20 kDa) than with oleoglobulin derived from oil tea seeds (15–16 kDa) after Maillard reaction coupling. CLSM further supports these findings, showing that higher molecular weight dextrans reduce droplet coalescence due to greater steric hindrance and enhanced surface charge, preventing aggregation and improving stability. Higher dextran molecular weights compared to protein molecular weights resulted in less effective emulsification. Therefore, it is hypothesized that coupling with smaller dextran molecular weights compared to protein molecular weights leads to better emulsification post-Maillard reaction. Overall, the increased molecular weight of dextran enhances internal interactions, contributing to more robust emulsification performance and system stability.

### 3.5. Antioxidant Activity

**Figure 12:**
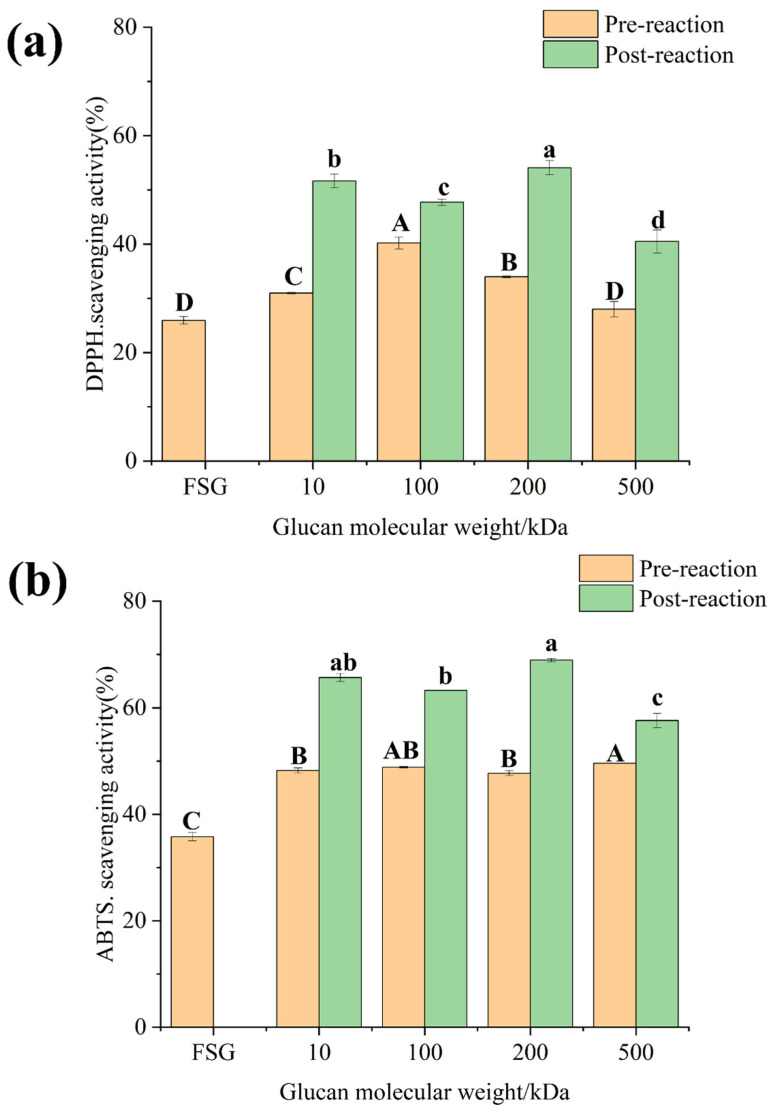
(**a**) DPPH and PDI; (**b**) ABTS (FSG: fish skin gelatin; 10, 100, 200, 500: corresponding molecular weights of dextran, respectively). Different lowercase letters indicate significant differences in post-reaction among different samples (*p* < 0.05), and different capital letters indicate significant differences in pre-reaction among different samples (*p* < 0.05).

As shown in Figure 12a, the DPPH radical scavenging activity of the conjugates significantly increases after the Maillard reaction compared to before the reaction. This enhancement has been previously demonstrated in earlier experiments (Tang, Wu [64]). Heat-treated proteins have been shown to exhibit decreased antioxidant activity due to oxidation. However, compared with the glycosylation products, the overall antioxidant activity of the products after the reaction actually increases [65]. This increase in antioxidant activity may be attributed to the formation of certain intermediate compounds that are formed during the Maillard reaction, which can provide hydrogen atoms. These low molecular weight intermediates, such as furans, can break the free radical chain and ultimately enhance the antioxidant activity [66]. After the reaction, the molecular weights of the glucans increased sequentially from 30.97%, 40.19%, 33.97%, and 28% to 51.64%, 47.73%, 54.07%, and 40.5%. The ABTS assay demonstrates that the reactants can quench free radicals either through hydrogen atom transfer mechanisms or by directly reducing them via electron transfer. Figure 12b provides evidence that the glycosylation products may act as hydrogen donors and exhibit strong ABTS cation radical scavenging activity [67].

## 4. Conclusions

This study demonstrated that glycosylation modification of fish skin gelatin (FSG) with dextran is an effective strategy for enhancing emulsion stability. The grafting of dextran onto FSG significantly increased its amphiphilicity, resulting in heightened hydrophobicity on the FSG surface due to covalent bonding with the glycoside. The incorporation of larger dextran molecules led to increased intermolecular repulsion compared with unmodified FSG, facilitating stronger adsorption at the oil–water interface and the formation of a denser protective layer. This advancement markedly improved the stability and emulsifying activity of the covalently coupled system. A positive correlation was observed between the molecular weight of dextran and the degree of covalent grafting, indicating that higher molecular weights enhance the modification process. Additionally, the introduction of polysaccharide chains altered the spatial organization and conformation of FSG, further contributing to its functional properties.

Moreover, the potential integration of Litsea cubeba essential oil into these glycosylated emulsions offers additional advantages, including enhanced flavor and antioxidant properties. Future investigations should focus on the application of these emulsions in the preservation of grilled fish, evaluating their effectiveness in extending shelf life and maintaining quality. Overall, the findings highlight the efficacy of the glycosylation reaction under dry Maillard conditions in modifying FSG, thereby expanding its value and potential applications within the food industry.

## Figures and Tables

**Table 1:** The secondary structure contents in FSG samples were identified by FTIR.

Dex	Pre/Post	Secondary Structure %				
		β-Folding	Irregularly Curled	α-Helix	β-Corner	β-Antiparallel
FSG	/	40.62 ± 0.36 ^Da^	24.48 ± 0.41 ^Ab^	/	20.66 ± 0.28 ^Ab^	14.41 ± 0.67 ^Ab^
10 K	Pre	41.25 ± 2.38 ^Da^	23.80 ± 0.01 ^Ab^	/	20.57 ± 1.66 ^Ab^	14.36 ± 0.72 ^Ac^
	post	64.61 ± 1.02 ^Ca^	/	21.61 ± 0.11 ^Ab^	10.05 ± 0.91 ^BCc^	3.71 ± 0.23 ^Cd^
100 K	Pre	40.84 ± 0.45 ^Da^	24.38 ± 0.25 ^Ab^	/	20.80 ± 0.55 ^Ab^	13.96 ± 0.35 ^Ac^
	post	71.56 ± 1.51 ^BCa^	/	19.23 ± 0.09 ^Bb^	6.98 ± 1.09 ^Cc^	2.21 ± 0.23 ^Dd^
200 K	Pre	47.43 ± 0.96 ^Da^	23.31 ± 1.54 ^Ab^	/	17.45 ± 0.60 ^Ac^	11.80 ± 0.02 ^Bd^
	post	77.16 ± 3.16 ^ABa^	/	15.50 ± 2.58 ^Cb^	5.39 ± 0.64 ^Cc^	1.94 ± 0.05 ^Dcd^
500 K	Pre	41.78 ± 2.00 ^Da^	24.07 ± 0.58 ^Ab^	/	20.06 ± 1.12 ^Ab^	14.07 ± 1.46 ^Ac^
	post	79.34 ± 1.49 ^Aa^	/	14.89 ± 0.6 ^Cb^	4.11 ± 0.84 ^Cc^	1.65 ± 0.04 ^Dd^

Notes: A–D, representing vertical difference comparisons in order of small to large, are represented by capital letters. Different letters denote different significance (*p* < 0.05). Horizontal difference comparisons are represented by lowercase letters, a through d, arranged from small to large.

## Data Availability

The original contributions presented in this study are included in the article. Further inquiries can be directed to the corresponding author.
