# Correction: Xing et al. Enhancing the Stability of Litsea Cubeba Essential Oil Emulsions Through Glycosylation of Fish Skin Gelatin via Dry Maillard Reaction. *Foods* 2024, *13*, 3847

**DOI:** 10.3390/foods14101741

**Published:** 2025-05-14

**Authors:** Naiwen Xing, Shikang Tang, Xuejiao Wang, Chaofan Guo, Xiaosong Hu, Junjie Yi

**Affiliations:** 1 Faculty of Food Science and Engineering, Kunming University of Science and Technology, Kunming 650500, China; 2 International Green Food Processing Research and Development Center of Kunming City, Kunming 650500, China; 3 Yunnan Key Laboratory of Plateau Food Advanced Manufacturing, Kunming 650500, China; 4 College of Food Science and Nutritional Engineering, China Agricultural University, Beijing 100083, China

Error in Figure

In the original publication [1], there was a mistake in Figure 10 as published. This data error occurred due to an unforeseen mistake during the data organization process. The corrected Figure 10 appears below. The authors state that the scientific conclusions are unaffected. This correction was approved by the Academic Editor. The original publication has also been updated.

## Figures and Tables

**Figure 10:**
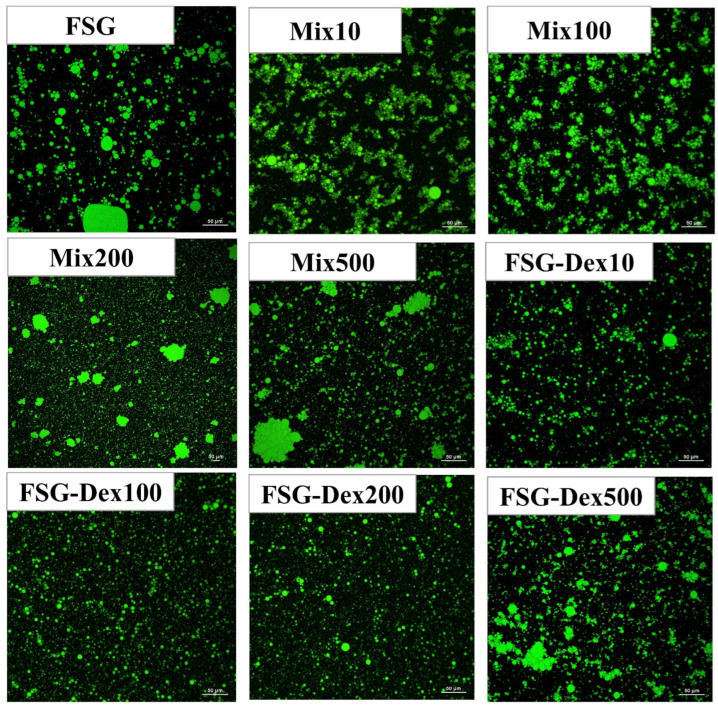
CLSM (FSG: fish skin gelatin; Mix10, Mix10, Mix100, Mix200, Mix500: mixtures of different molecular weights; FSG–Dex10, FSG–Dex100, FSG–Dex200, FSG–Dex10, FSG–Dex100, FSG–Dex200, FSG–Dex500: couplings of different molecular weights.).
